# A novel film based on a cellulose/sodium alginate/gelatin composite activated with an ethanolic fraction of *Boswellia sacra* oleo gum resin

**DOI:** 10.1002/fsn3.3819

**Published:** 2023-12-07

**Authors:** Saurabh Bhatia, Yasir Abbas Shah, Ahmed Al‐Harrasi, Sana Ullah, Md Khalid Anwer, Esra Koca, Levent Yurdaer Aydemir, Mahbubar Rahman Khan

**Affiliations:** ^1^ Natural and Medical Sciences Research Center University of Nizwa Nizwa Oman; ^2^ School of Health Science University of Petroleum and Energy Studies Dehradun India; ^3^ Saveetha Institute of Medical and Technical Sciences Saveetha University Chennai India; ^4^ Department of Pharmaceutics, College of Pharmacy Prince Sattam Bin Abdulaziz University Al‐kharj Saudi Arabia; ^5^ Department of Food Engineering, Faculty of Engineering Adana Alparslan Turkes Science and Technology University Adana Turkey; ^6^ Department of Food Processing and Preservation Hajee Mohammad Danesh Science & Technology University Dinajpur Bangladesh

**Keywords:** cellulose, food packaging, gelatin, plant extract, sodium alginate

## Abstract

*Boswellia sacra* and its derivatives exhibit notable bioactive properties, which have been the subject of extensive scientific research; however, their potential applications in food packaging remain largely untapped. In the current study, cellulose, sodium alginate, and gelatin composite edible films were fabricated with the addition of different concentrations (0.2% and 0.3%) of the ethanolic fraction of *Boswellia sacra* oleo gum resin (BSOR). The resultant films were examined for their physical, chemical, mechanical, barrier, optical, and antioxidant properties. Moreover, the films were characterized using Scanning Electron Microscopy (SEM), X‐ray diffraction (XRD), and Fourier transform infrared spectroscopy (FTIR) to study the impact of incorporating BSOR on the morphological, crystalline, and chemical properties of the films. The addition of BSOR increased the film thickness (0.026–0.08 mm), water vapor permeability (0.210–0.619 (g.mm)/(m^2^.h.kPa), and the intensity of the yellow color (3.01–7.20) while reducing the values of both tensile strength (6.67–1.03 MPa) and elongation at break (83.50%–48.81%). SEM and FTIR analysis confirmed the interaction between the BSOR and film‐forming components. The antioxidant properties of the edible films were significantly increased with the addition of BSOR. The comprehensive findings of the study demonstrated that BSOR possesses the potential to serve as an efficient natural antioxidant agent in the fabrication of edible films.

## Graphical abstract

Cellulose, sodium alginate, and gelatin composite edible films were fabricated with the addition of different concentrations of the ethanolic fraction of *Boswellia sacra* oleo gum resin (BSOR). The resultant films showed great potential as a food packaging material.

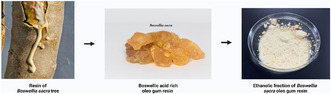

## 1 INTRODUCTION

The primary purpose of food packaging is to serve as a barrier between the food and the external environment, thereby reducing or preventing exposure to food deteriorating factors like oxygen, moisture, and microorganisms. Edible films reduce plastic waste and promote a more eco‐friendly and sustainable approach to food packaging. The scientific investigations within the domain of edible films revolve around altering the formulation of the film‐forming solution, integrating antimicrobial and antioxidant substances, and utilizing food waste as a source to extract biopolymers for their application as sustainable food packaging material (Rai et al., 2019). *Boswellia sacra*, also known as Frankincense or Olibanum, is a type of resin that has been traditionally used for its antioxidant and anti‐inflammatory properties (Ammon, 2006; Ernst, 2008). In *Boswellia sacra*, the resinous component contains various compounds, including monoterpenes, diterpenes, triterpenes, tetracyclic triterpenic acids, and four prominent pentacyclic triterpenic acids. These four major pentacyclic triterpenic acids, namely β‐boswellic acid, acetyl‐β‐boswellic acid, 11‐keto‐β‐boswellic acid, and acetyl‐11‐keto‐β‐boswellic acid, are the primary components responsible for its biological activities (Siddiqui, 2011).

Previous research has shown the benefits of adding Frankincense extract to biopolymer‐based films for potential food packaging applications. (Narasagoudr et al., 2020) reported a significant increase in the mechanical, barrier, antimicrobial, and morphological properties of the chitosan and polyvinyl alcohol‐based composite films when incorporated with varying concentrations of boswellic acid. In another study, the addition of boswellic acid to polyvinyl alcohol‐based films resulted in improved mechanical and barrier properties (Narasagoudr et al., 2020).

The characteristics of composite biopolymer‐based films indicate a high degree of compatibility and stability, resulting in decreased brittleness and increased resilience to various environmental factors. Cellulose, sodium alginate, and gelatin have vast applications in the preparation of edible films and coating materials to enhance the safety and quality of food products. Cellulose, with its readily accessible, sustainable, and biocompatible nature, possesses unique characteristics that position it as a pivotal resource for promoting sustainability on our planet in the future (Singh et al., 2019). Sodium alginate functions as a low‐cost, eco‐friendly, biocompatible, and non‐toxic hydrocolloid. This substance has applications in multiple domains, including its function as a thickener, its ability to form gels, and its capacity to serve as a colloidal stabilizer within the beverage industry (Gheorghita et al., 2020). Moreover, a substantial body of research has been undertaken to investigate the film‐forming characteristics of gelatin as well as its favorable functional characteristics and efficacy as a protective outer layer for food items, protecting against moisture loss and oxidation (Suderman et al., 2018). A composite film can be developed by blending cellulose, sodium alginate, and gelatin, thereby integrating the beneficial properties of each constituent.

Various research studies have highlighted the promising potential of incorporating bioactive compounds, plant extracts, and essential oils into edible films and their profound impact on their physicochemical properties (Chaari et al., 2022; Moosavi‐Nasab et al., 2023; Smaoui et al., 2022; Yadav et al., 2022). There are no or very limited studies available in the literature that comprehensively investigate the utilization of bioactive compounds extracted from *Boswellia sacra* in the development of edible films as natural antioxidant agents. Therefore, the objective of the current study is to examine the effect of the incorporation of BSOR in composite films based on cellulose, sodium alginate, and gelatin blends for potential food packaging applications. Furthermore, this study aims to evaluate the mechanical, barrier, optical, antioxidant, antimicrobial, and morphological characteristics of the resultant films.

## 2 MATERIALS AND METHODS

### 2.1 Chemical procurement

Cellulose, sodium alginate, and gelatin were procured from Sisco Research Laboratories Pvt. Ltd., situated in Mumbai, India. Additionally, key chemical compounds, including 2,2′‐diphenyl‐1‐picrylhydrazyl (DPPH), ABTS (2,2′‐azinobis‐(3‐ethylbenzothiazoline‐6‐sulfonic acid)) and Trolox (6‐Hydroxy‐2,5,7,8‐tetramethylchromane‐2‐carboxylic acid), were sourced from Sigma‐Aldrich, headquartered in St. Louis, MO, USA.

### 2.2 Extraction of the ethanolic fraction of *Boswellia sacra* oleo gum resin

**FIGURE 1:**
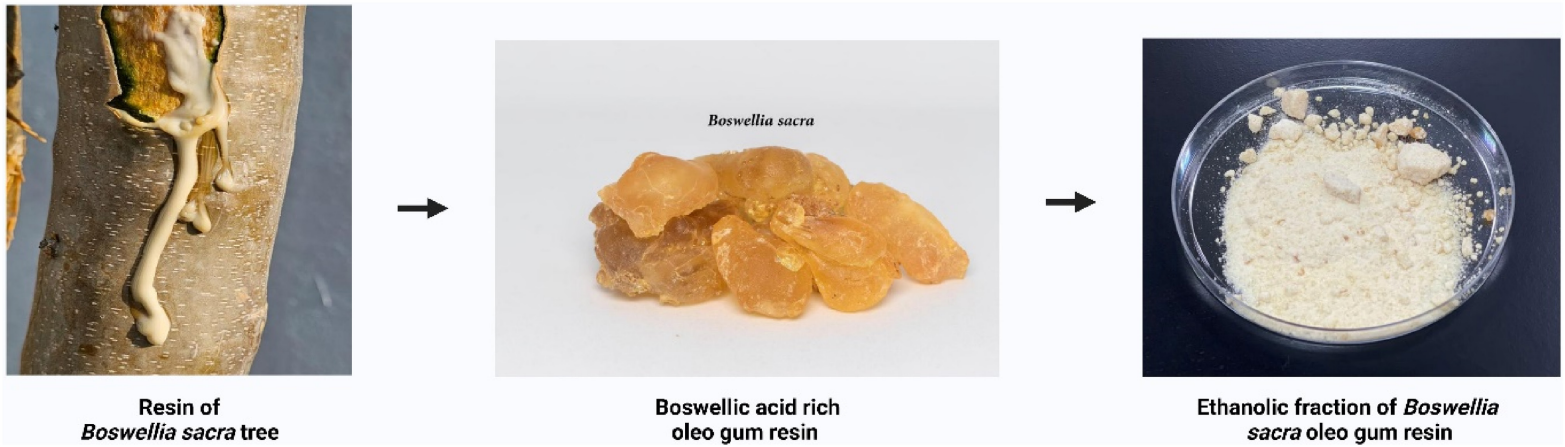
Graphical representation of the extraction of the ethanolic fraction of *Boswellia sacra* oleo gum resin.

The extraction of boswellic acid‐rich oleo gum resin was carried out as per the procedure outlined in a previous study (Jauch & Bergmann, 2003). Initially, the resin of the *Boswellia sacra* tree was hydrodistilled to remove the essential oil. After removing the essential oil, the spent residue was extracted with ethanol to get the boswellic acid‐rich oleo gum resin. Boswellic acid‐rich oleo gum resin was extracted with a 0.5 *N* NaOH solution. The diluted NaOH layer was acidified with 2 *N* HCl to get the white, amorphous precipitates. The amorphous ppt was dried under a vacuum to get the Boswellic acid (70%) powder (Figure 1).

### 2.3 Film samples preparation

The film‐forming solutions were prepared by following the casting method as described in our previous study (Bhatia, al‐Harrasi, Shah, Altoubi, et al., 2023). Initially, 1% (w/v) solutions of cellulose, sodium alginate, and gelatin were prepared separately in distilled water and continuously stirred for 2 h at room temperature. After complete dissolution of the polymers in distilled water, all the solutions were mixed, and then glycerol 0.5% (v/v) was added as a plasticizer and continuously mixed at room temperature for 30 minutes. The ratio of the polymers (cellulose, sodium alginate, and gelatin) was 60:20:20, respectively, in a 20 mL solution mixture. After the preparation of the film‐forming solution, BSOR was added to the mixture in 0.2% and 0.3% concentrations. The prepared solutions were subsequently transferred onto plastic petri plates and subjected to air‐drying at ambient conditions. Once thoroughly dried, the films were carefully peeled from the plates and subsequently processed for further analysis. A detailed description of film‐forming composition is provided in Table 1.

**TABLE 1:** Detailed chemical composition of BSOR‐containing films.

Sample code	Biopolymers (w/v)	Plasticizer (glycerol) (v/v)	BSOR (w/v)
CO‐1/Blank	CS 1% + SA 1% + GE 1%	0.5%	N/A
CO‐2	CS 1% + SA 1% + GE 1%	0.5%	0.2%
CO‐3	CS 1% + SA 1% + GE 1%	0.5%	0.3%

Abbreviations: CS, Cellulose; GE, Gelatin; SA, Sodium Alginate.

### 2.4 Thickness

The thickness of the composite film samples was evaluated using a digital micrometer (Yu‐Su 150, Yu‐Su Tools) following established protocols. Five random measurements were conducted at distinct points on each film sample, and the average value in (mm) was computed.

### 2.5 Mechanical properties

The mechanical characteristics of the films were assessed following the ASTM D882 standard method, a well‐accepted procedure by the American Society for Testing and Materials (ASTM, 2010). The testing apparatus consisted of a Universal Tester (TA.XT plus, Stable Micro Systems, England) equipped with a 5 kg load cell. Before conducting the tests, the films were conditioned for a minimum of 40 h in a test cabinet (Nüve TK 120, Türkiye) at 50% relative humidity and 25 °C. To determine the mechanical properties, strips of the films, measuring 7 mm in width and 60 mm in length, were subjected to analysis at a testing speed of 30 mm/min. Two key parameters, including tensile strength (TS) in units of MPa and elongation at break (EAB) as a percentage, were calculated automatically by the Exponent Connect software platform.

### 2.6 Water solubility and moisture percentage

Water solubility analysis was carried out according to a modified testing method originally introduced by (Kim & Song, 2018). The film strips (3 × 4 cm^2^) underwent a drying procedure in an oven maintained at 105°C until a constant weight was attained. The resulting weight, denoted as W1, was subsequently documented. Subsequently, the film strip was mixed with 20 mL of deionized water and placed in a shaking incubator for 24 h. Then, the film was extracted from the water and subjected to drying in an oven set at 105°C. Once the drying process was completed, the weight of the film was measured and recorded as W2. The solubility of films in water was measured using the equation provided below.

(1) $$\mathrm{WS}=\frac{W1-W2}{W1}\times 100$$

The gravimetric approach was utilized to ascertain the MC of the edible films. The weight of the film strips that were cut into measurements of 3 cm by 4 cm was recorded as W1. After that, the films were dried at 105°C until they attained a consistent weight, and the weight of the dried film was recorded as W2. The following equation was utilized to calculate the MC of each of the films.

(2) $$\mathrm{MC}=\frac{W1-W2}{W1}\times 100$$

### 2.7 Water barrier properties

The determination of the water vapor permeability (WVP) of the films involved quantifying weight variations through a technique elucidated by (Erdem et al., 2019). The experimental procedure comprised subjecting the films to a controlled environment within a desiccator, where the humidity was maintained at 50%. Glass test cups with a diameter of 5 cm and a depth of 3 cm were used for the experiment. The humidity levels in the measurement system were controlled by adding water (100% humidity) and silica gel (0% humidity). The films were tightly sealed on top of the cup containing silica gel, and the cups were weighed periodically every hour throughout the day to assess any weight increase. The WVP values were then calculated using the following equation:

(3) $$\mathrm{WVP}=\frac{\mathrm{\Delta m}}{\mathrm{\Delta t}\times \mathrm{\Delta P}\times A}\times d$$

WVP is represented in units of gmm/(m^2^)(d)(kPa). Within this formula, the variable ∆m/∆t symbolizes the rate of gaining moisture, denoted in grams per day. The parameter A embodies the surface area of the membrane in square meters. The term ∆P signifies the disparity in aqueous vapor pressure spanning the two facets of the membrane, calculated in kilopascals. Lastly, the variable d characterizes the thickness of the membrane in millimeters.

### 2.8 Optical and color properties

To assess the chromatic characteristics of the film samples, a colorimeter manufactured by Konica Minolta in Tokyo, Japan, was used. This device is designed to precisely measure color properties and was employed to assess the L*, a*, and b* parameters. To ensure a comprehensive assessment of color, multiple points across the film surface were analyzed. This approach allowed for a more precise representation of the overall color characteristics of the films. The overall color variation was determined using the following equation.

(4) $${\mathrm{\Delta E}}^{*}={\left[{\left({\Delta L}^{*}\right)}^{2}+{\left({\Delta a}^{*}\right)}^{2}+{\left({\Delta b}^{*}\right)}^{2}\right]}^{1/2}$$

The parameters used to analyze and quantify color characteristics in this equation include L (lightness), a* (green‐red color), b* (blue‐yellow color), and ΔE* (overall color variation).

The transparency of the films was assessed utilizing a spectrophotometric technique employing an ONDA‐Vis spectrophotometer, following the protocol outlined by (Erdem et al., 2019). To measure transparency, rectangular films were positioned within Spectro cuvettes, and their light transmission was assessed at a specific wavelength of 550 nm using the spectrophotometer. Subsequently, the transparency of each film was determined employing the prescribed equation.

(5) $$\text{Transparency}=\left(\frac{A550}{X}\right)$$

### 2.9 Antioxidant activity

The films underwent assessment for their antioxidative capacity using two approaches: ABTS^•+^ and DPPH radical scavenging methods. The evaluation of ABTS^•+^ radical binding ability was conducted following a technique described by (Re et al., 1999) with some modifications. The film samples were mixed with a solution containing ABTS^•+^ radicals, and the change in absorbance at 734 nm was monitored for 6 minutes. This measurement was taken after vigorously mixing the film samples with a specified quantity of film and the ABTS^•+^ radical solution. The ABTS^•+^ radical solution had a concentration of 7 mmol/L and was prepared using a potassium persulfate solution (2.45 mM).

To assess the scavenging capability of the films against DPPH (2,2‐diphenyl‐1‐picrylhydrazyl) free radicals, a technique outlined by (Brand‐Williams et al., 1995) was employed. In accordance with this methodology, 50 mg of film specimens were mixed with 1.95 mL of a DPPH solution, the concentration of which was adjusted to achieve an absorbance value of 0.7 ± 0.2 at a wavelength of 517 nm. After incubation, the absorbance of the solution was quantified using an ONDA Visible (Vis) spectrophotometer at a wavelength of 517 nm. The results of antioxidant activity were quantified as a percentage of inhibition and were derived from the mean of three independent measurements.

### 2.10 Microstructure

The morphological properties of the prepared film samples were analyzed utilizing a JSM6510LA Analytical Scanning Electron Microscopy (SEM) apparatus manufactured by Jeol, a company located in Tokyo, Japan. The examination followed the methodology specified in our prior research (Bhatia, al‐Harrasi, Jawad, Shah, et al., 2023).

### 2.11 FTIR analysis

In this investigation, the assessment of chemical interactions occurring within the film matrix was conducted using an Infrared Tensor 37 instrument (InfraRed Bruker, Ettlingen, Germany). The apparatus was set up to perform 32 scans across a spectral range of 400–4000 cm^−1^. By employing this setup, a thorough examination of the film's constituents was facilitated, enabling the identification and analysis of their respective chemical interactions.

### 2.12 XRD analysis

The extent of crystalline nature in the film samples was evaluated through X‐ray diffraction employing a Bruker D8 Discover instrument equipped with copper (Kα) radiation (λ = 1.5418 Å) and operated at 40 kV. The examination encompassed an angular range of 2θ = 5°–θ = 55°, with a data collection rate of 0.500 s/point.

### 2.13 Statistical analysis

The obtained results were reported as the mean ± standard deviation (SD) of three replicates and were subjected to statistical analysis using a software package for data analysis (SPSS ver. 17.0; SPSS Inc). To assess the significance of the differences observed among the means, a one‐way analysis of variance (ANOVA) was conducted. The statistical analysis employed Duncan's test at a predetermined significance level of 5%. By using this statistical approach, the study was able to determine if there were any significant variations between the means.

## 3 RESULTS AND DISCUSSION

### 3.1 Visual analysis of the films

**FIGURE 2:**
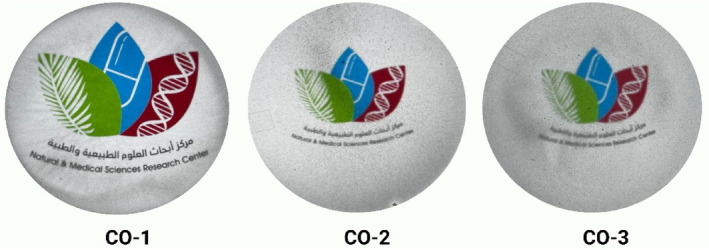
Visual analysis of the cellulose/sodium alginate/gelatin composite films loaded with and without BSOR.

In the visual observation, as shown in Figure 2, the CO‐1 film sample exhibited more transparency and homogeneous structure as compared to the CO‐2 and CO‐3 film samples. The observed difference in transparency indicates that the incorporation of BSOR into the composite films affected their optical properties. It was observed that BSOR‐loaded films exhibited enhanced adhesion to the petri plate surface, displaying higher resistance to peeling compared to the control (blank) films. Furthermore, the BSOR‐loaded films demonstrated increased fragility in comparison to the blank films. The visual analysis revealed that the incorporation of BSOR into the polymer matrix led to an increase in opacity and surface roughness.

### 3.2 Thickness

Thickness values obtained for film samples based on cellulose, sodium alginate, and gelatin are shown in Table 2. The addition of BSOR to the film sample significantly (*p* < .05) enhanced the thickness from 0.026 to 0.08 mm. Most of the plant extracts resulted in an increase in the film thickness, potentially attributed to a greater concentration of solids incorporated within the film structure (Kola, 2020). Several plant extracts incorporated films based studies showed the similar findings (Riaz et al., 2020; Yuan et al., 2020; Zhang, Lian, et al., 2020).

**TABLE 2:** Thickness, mechanical, and barrier properties of the edible films based on cellulose, sodium alginate, and gelatin composites loaded with and without BSOR.

Film sample	Thickness (mm)	TS (MPa)	EAB (%)	WVP ((g*mm)/(m^2^*h*kPa))	MC
CO‐1	0.026 ± 0.005^a^	6.67 ± 0.59^a^	83.50 ± 3.94^a^	0.210 ± 0.01^a^	37.96 ± 1.34^a^
CO‐2	0.052 ± 0.008^b^	1.38 ± 0.13^b^	57.42 ± 3.74^b^	0.417 ± 0.02^b^	36.29 ± 1.45^ab^
CO‐3	0.08 ± 0.007^c^	1.03 ± 0.15^c^	48.81 ± 5.41^c^	0.619 ± 0.02^c^	34.93 ± 0.76^b^

*Note*: The ± sign means standard deviations. Column values followed by the letters (a, b, and c) are significantly different (*p* < .05).

### 3.3 Mechanical attributes

The mechanical characteristics indicate how well the films can safeguard the physical structure of food (Rubilar et al., 2013). The impact of adding different amounts of BSOR on the mechanical properties of a composite film made of cellulose, sodium alginate, and gelatin is shown in Table 2. The CO‐1 film sample, which did not contain BSOR, exhibited a higher tensile strength (TS) of 6.67 MPa and an elongation at break (EB) of 83% as compared to BSOR‐loaded films. The incorporation of BSOR in CO‐2 and CO‐3 film samples significantly (*p* < .05) decreased the TS and EAB. With the inclusion of BSOR and an increase in its concentration, the TS dropped significantly from 6.67 to 1.03 MPa, while the EAB decreased from 83% to 48%. The possible reason for the decline in TS and EAB may be attributed to the decrease in molecular bonding between the polymer strands and BSOR.

Previous studies have reported similar results in which the addition of plant extracts decreased the tensile strength as well as the elongation at break of the biopolymer‐based films.A previous study by (Zhang, Lian, et al., 2020) reported that the addition of natural plant extracts such as pine nutshell, peanut shell, and winter jujube leaf to chitosan films resulted in a reduction in both TS and EAB of the resultant films. In a previous study, (Kalkan et al., 2020) examined the impact of *Rheum ribes* extract on the TS and EAB values of methylcellulose‐based films. The inclusion of the extract resulted in increased fragility of the films. Simultaneously, the films exhibited decreased values in terms of elongation at break, tensile strength, durability, and elasticity (Kalkan et al., 2020). The results obtained from the current study demonstrate that a lower concentration of BSOR is suitable for producing films with desirable properties.

### 3.4 Water vapor permeability and moisture content

Edible films primarily serve the purpose of impeding moisture transfer between food and the surrounding atmosphere, thereby improving food product safety and extending shelf life. Table 2 illustrates the influence of incorporating BSOR on the WVP of the cellulose, sodium alginate, and gelatin‐based composite films. CO‐1 or blank film sample exhibited a lower WVP as compared to film samples containing various concentrations of the BSOR. With the introduction of BSOR into the film matrix, the WVP of the films increased significantly (*p* < .05). Notably, the CO‐3 film sample, containing the highest BSOR concentration (0.3%), exhibited the maximum WVP values. This observed trend can be attributed to structural alterations in the cellulose, sodium alginate, and gelatin‐based film matrix due to the addition of BSOR. The presence of BSOR induced changes in the molecular arrangement of the film network, resulting in an increase in free volume within the structure. Consequently, the film network became less dense, leading to enhanced permeability. (Chana‐Thaworn et al., 2011) observed comparable results, indicating that the incorporation of kiam wood (*Cotyleobium lanceotatum*) extract led to a notable rise in the WVP of films based on hydroxypropyl methylcellulose. Another research conducted also resulted in comparable findings, indicating that the WVP measurements of the soy protein isolate‐based films exhibited an increase when Mexican oregano extract (*Lippia graveolens*) was incorporated (Pruneda et al., 2008).

Table 2 provides the findings regarding the impact of incorporating BSOR on the moisture content of composite films made from cellulose, sodium alginate, and gelatin. The moisture content of the CO‐1/blank film sample was significantly higher at 37.96% compared to the moisture content of the CO‐2 and CO‐3 film samples loaded with BSOR. As the concentration of BSOR increased from 0.2% to 0.3%, there was a noticeable reduction in moisture content. This decrease can be attributed to the interaction between biopolymers and BSOR at the molecular level. Similar results were also presented by other researchers, in which the incorporation of extract decreased the moisture content of the film (Kaewprachu et al., 2017; Nguyen et al., 2020; Nouri et al., 2018).

### 3.5 Optical and color properties

Plant extracts are commonly used to produce films that possess a higher opacity in comparison to films without these extracts. When plant extracts are incorporated into films, they create a sufficient shield against light, which is crucial for safeguarding light‐sensitive components from degradation (Kola, 2020). Table 3 shows the color properties and transparency of composite films made from cellulose, sodium alginate, and gelatin with varying concentrations of BSOR. Incorporating BSOR into the films had a notable impact on both color and transparency. As the concentration of BSOR in the film increased, the transparency of the film decreased significantly (*p* < .05). Additionally, the edible films with BSOR turned yellow, which was evident from the decreased L (lightness) and increased b (yellowness) values, as shown in Table 3. This color change could be attributed to the presence of phenolic acids and flavonoids in the BSOR. As the amount of BSOR increased, the overall color difference (∆E) of the films also increased significantly (*p* < .05). Similar findings were achieved in the investigations where the extracts were added into the edible films (Jutaporn et al., 2011; Yuan et al., 2020; Zhang, Li, & Jiang, 2020; Zhang, Lian, et al., 2020).

**TABLE 3:** Transparency and color attributes of the edible films based on cellulose, sodium alginate, and gelatin composites loaded with and without BSOR.

Film sample	Transparency	L	a*	b*	ΔE*
CO‐1	90.16 ± 0.45^a^	95.69 ± 0.31^a^	−0.02 ± 0.01^a^	3.01 ± 0.06^a^	2.91 ± 0.06^a^
CO‐2	28.42 ± 1.31^b^	94.18 ± 0.18^b^	−0.38 ± 0.07^b^	6.42 ± 0.19^b^	4.54 ± 0.13^b^
CO‐3	11.79 ± 0.65^c^	84.80 ± 0.70^c^	0.70 ± 0.01^c^	7.20 ± 0.26^c^	8.37 ± 0.68^c^

*Note*: The ± sign means standard deviations. Column values followed by the letters (a, b, and c) are significantly different (*p* < .05).

Abbreviations: a*, green‐red color; b*, blue‐yellow color; L, lightness; ΔE*, overall color variation.

### 3.6 Antioxidant activity of the films

**FIGURE 3:**
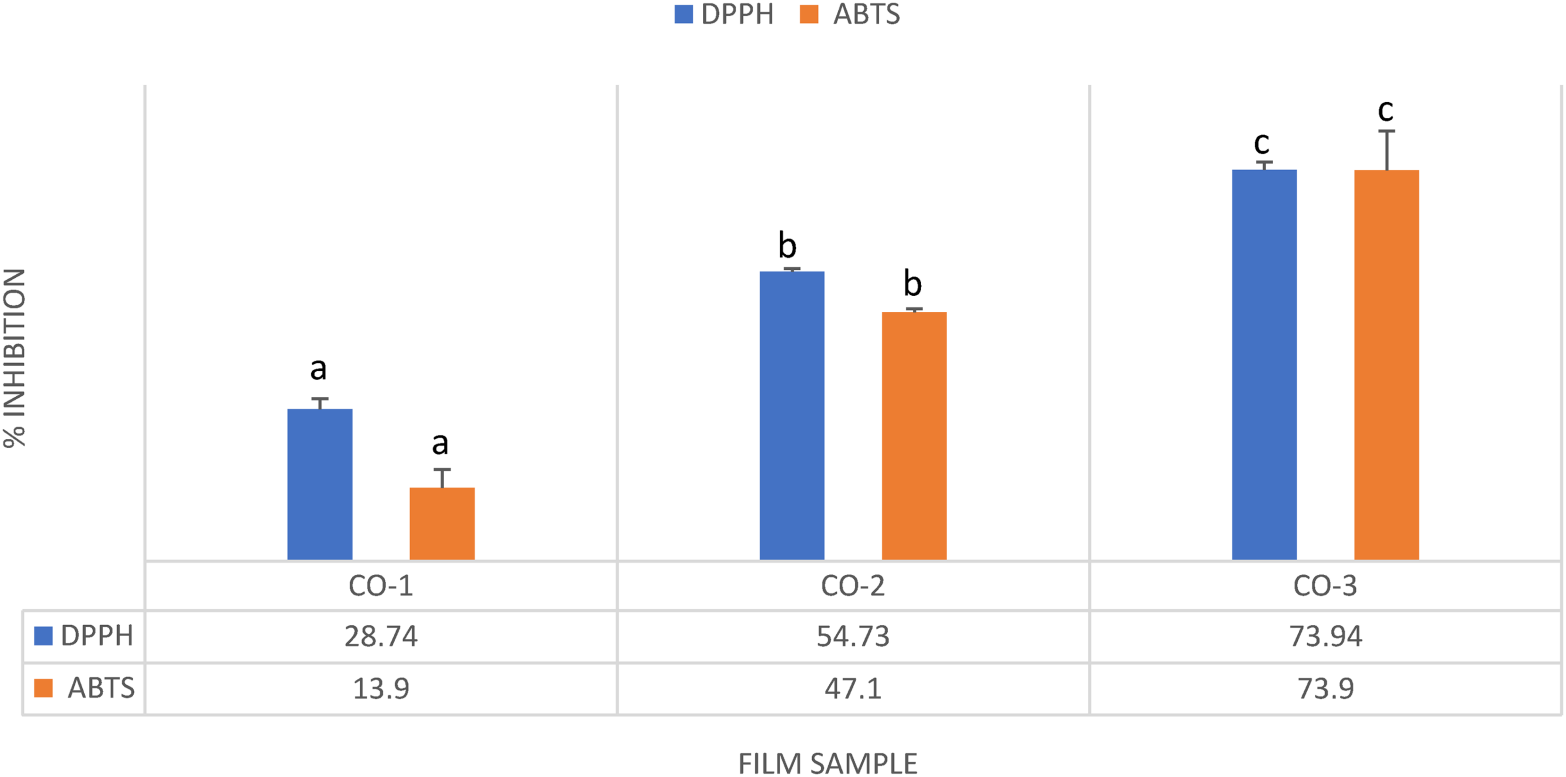
Antioxidant activity of the cellulose, sodium alginate, and gelatin‐based composite films loaded with and without BSOR. The letters above the columns (a, b and c) are significantly different (*p* < 0.05).

The DPPH and ABTS^•+^ radical scavenging activities of different film samples loaded with and without BSOR are shown in Figure 3. For the analysis of the antioxidant activity, 50mg of the film sample was utilized for DPPH, and 10mg for ABTS^•+^ radical scavenging activity. The inclusion of BSOR in cellulose, sodium alginate, and gelatin composite films led to a notable enhancement (*p* ≤ .05) in their antioxidant capacity. Specifically, CO‐2 and CO‐3 film samples exhibited a substantial increase in antioxidant activity compared to CO‐1 films. These findings suggest that the higher antioxidant capacity can be due to the presence of phenolic compounds in BSOR, which interact with and neutralize free radicals. A previous study found that incorporating tea polyphenols into gelatin and sodium alginate composite films resulted in enhanced antioxidant activity (Dou et al., 2018). In our previous study, the incorporation of the Ficus fruit aqueous extract significantly enhanced the radical scavenging activity of the films based on the chitosan and sodium alginate composite (Bhatia, al‐Harrasi, Shah, Jawad, et al., 2023). Many researchers have developed active packaging films that incorporate antioxidants derived from various agricultural sources, and their findings indicate similar results (Kam et al., 2018; Liu et al., 2019; Rodríguez et al., 2020; Thivya et al., 2021). Consequently, the addition of BSOR holds promise for enhancing the antioxidant potential of cellulose, sodium alginate, and gelatin‐based films, thereby expanding their applications in various industries.

### 3.7 Microstructure of the films

**FIGURE 4:**
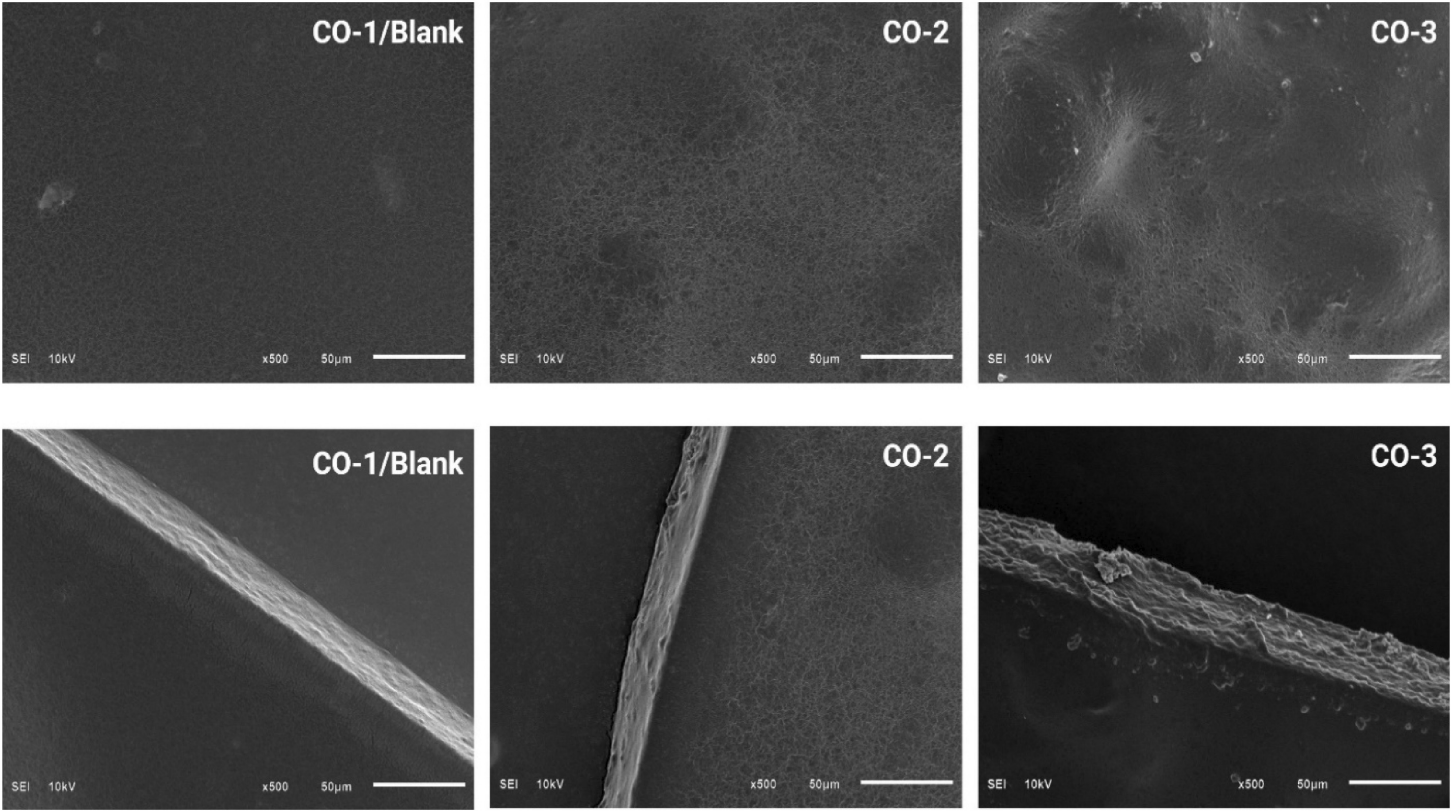
SEM images of the surface and cross‐sectional morphology of the cellulose, sodium alginate, and gelatin‐based composite films loaded with and without BSOR.

Figure 4 presents the SEM images of the prepared film samples with and without BSOR. The smooth and homogeneous surfaces of the blank or CO‐1 sample, which is composed of a cellulose/sodium alginate/gelatin composite, suggest strong intermolecular interaction between the biopolymers. A lower quantity of particles was observed on the film surface of CO‐1 samples in comparison to CO‐2 and CO‐3. The cross section of the films revealed a consistent and uniform structure without any irregularities such as pores or cracks in the blank film sample.

Incorporating BSOR into the film matrix led to variations in surface characteristics, where the presence of agglomerates and particles on the film surface was observed in the CO‐2 and CO‐3 samples. This indicates that the addition of BSOR resulted in surface heterogeneity in the film samples, as observed in Figure 4. Considering the aspects of film consistency, uniformity, and optical characteristics, it seems more suitable to produce films with a lower concentration of BSOR to maintain integrity, homogeneity, and desirable optical properties.

### 3.8 FTIR

**FIGURE 5:**
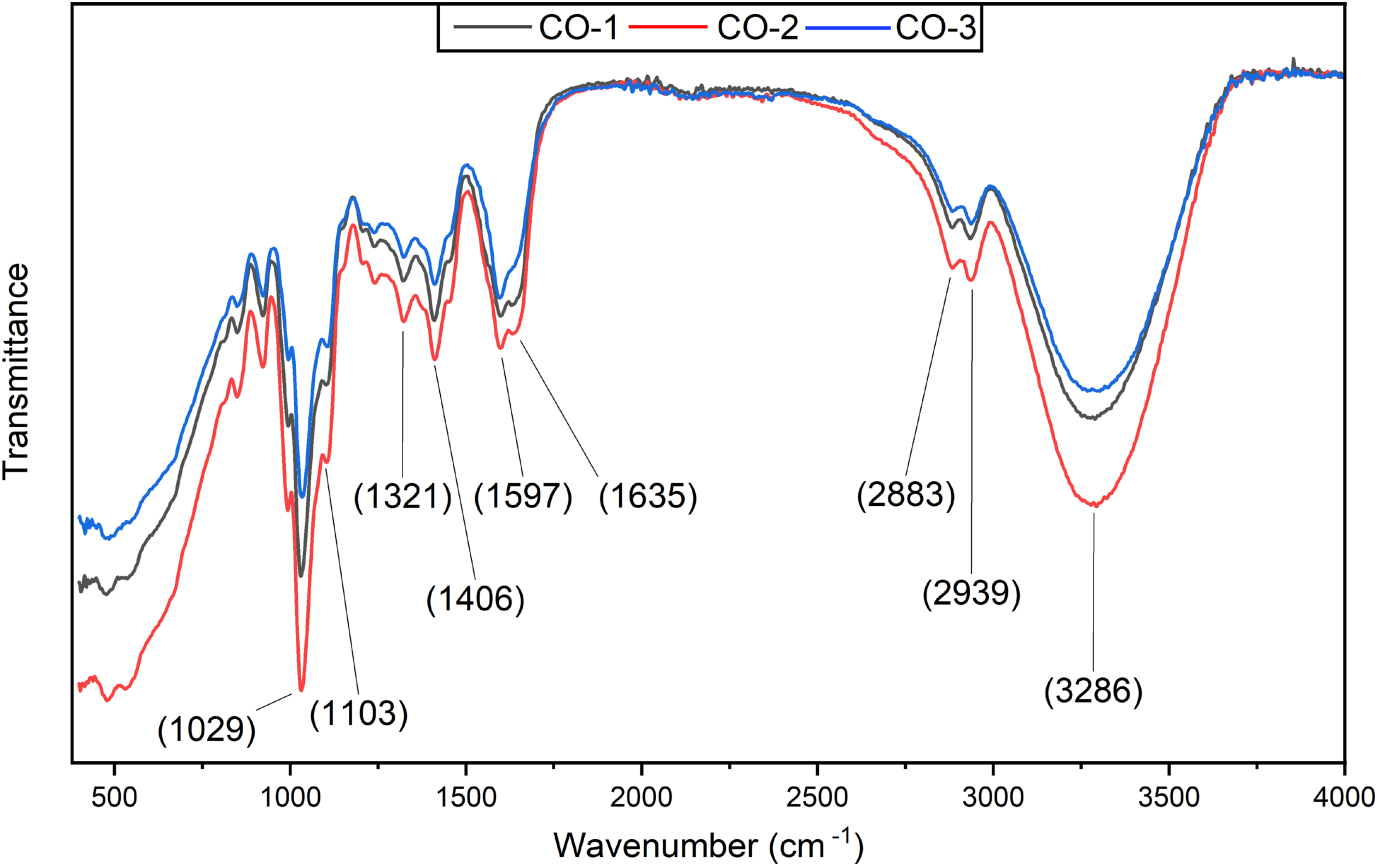
FTIR spectra of the cellulose, sodium alginate, and gelatin‐based composite films loaded with and without BSOR.

FTIR was employed to analyze the composite films based on the cellulose, sodium alginate and gelatin, focusing on the intermolecular bonding between the various functional groups present in the film components. The composite films showed bands at 3286, 2939, 2883, 1635, 1597, 1406, 1321, 1103, and 1029 cm^−1^ wavenumber positions, as shown in Figure 5. The band position at 3286 cm^−1^ is attributed to the presence of the ‐OH group, the band position at 2939 cm^−1^ corresponds to the stretching of the C‐H group, and the band position at 2883^−1^ corresponds to the stretching of the N‐H group. The band positions at 1635, 1597, and 1406 cm^−1^ correspond to medium stretching of the C=C group, medium bending of the N‐H group, and medium bending of the O‐H group, respectively. Similarly, band position at 1321 cm^−1^ is attributed to the medium bending of the O‐H group, and band positions at 1103 and 1029 cm^−1^ are attributed to the strong stretching of the C‐O group. Bands at 3286 and 1406 confirm the presence of sodium alginate (Fan et al., 2005). The characteristic band at 1597 cm^−1^ wavenumber position confirms the presence of gelatin in the polymer matrix (Chiaoprakobkij et al., 2019). Similarly, band positions at 1635 and 1029 cm^−1^ confirm the presence of cellulose in the polymer matrix (Barud et al., 2008; Chiaoprakobkij et al., 2019). The stretching of C‐H and N‐H groups at 2939 and 2883 cm^−1^ band positions is attributed to the cellulose‐alginate complexes (Salama et al., 2019). The change in peak position, with previous reports, could be due to the presence of multiple polymers or the addition of BSOR. The current FTIR results are in line with previously reported studies (Chiaoprakobkij et al., 2019, 2020; Dong et al., 2006; Salama et al., 2019).

### 3.9 XRD analysis

**FIGURE 6:**
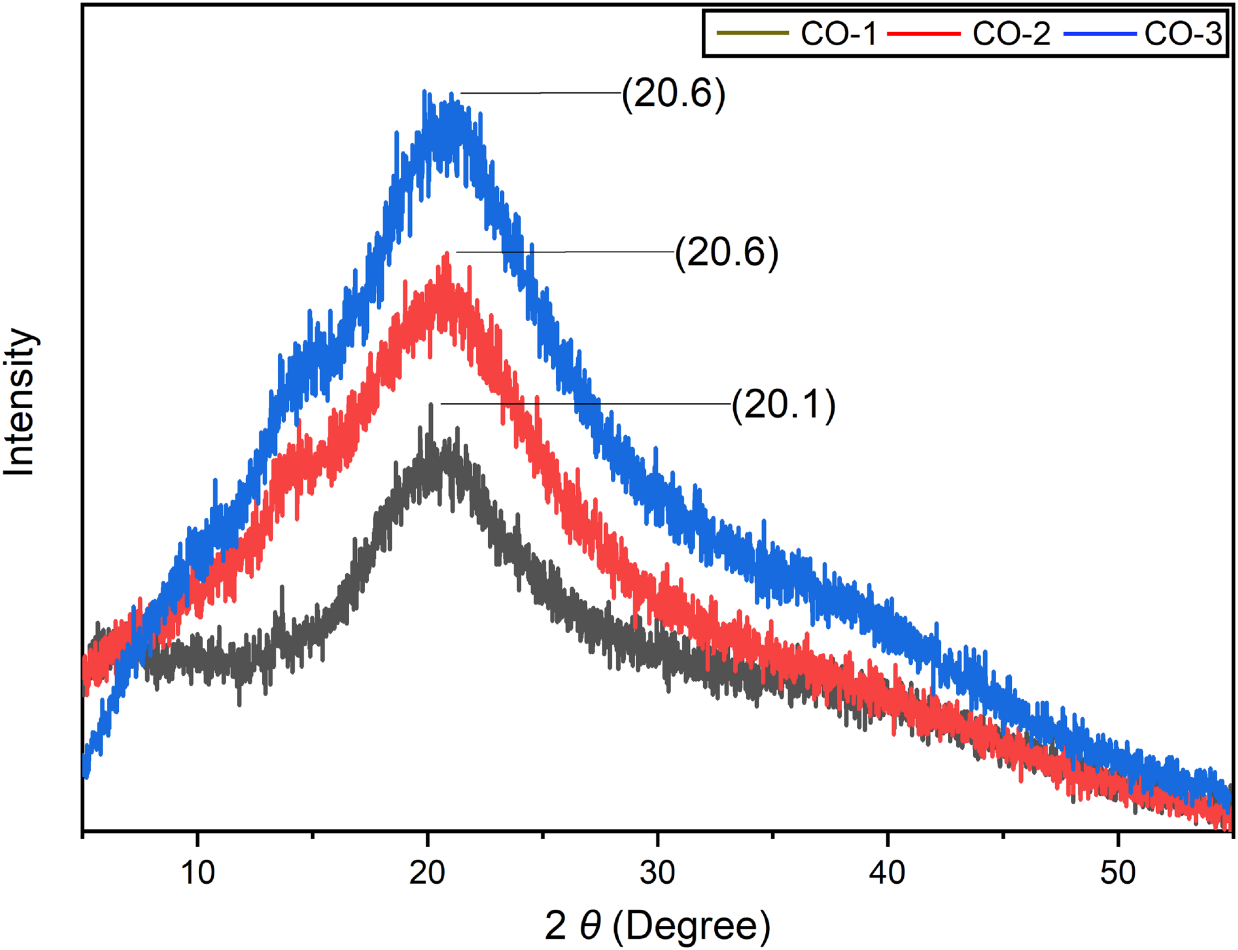
XRD chromatogram of cellulose, sodium alginate, and gelatin‐based composite films loaded with and without BSOR.

To assess the impact of BSOR on film crystallinity XRD investigation was performed. Films with a high degree of crystallinity exhibit a narrow and high peak shape, whereas films with lower crystallinity display a broader peak shape (Zhou et al., 2021). Figure 6 presents the X‐ray diffraction patterns obtained for cellulose, sodium alginate, and gelatin‐based composites before and after the addition of BSOR. The study focused on examining the X‐ray diffraction pattern, crystallinity, and changes in intensity resulting from the incorporation of BSOR. Using the Diffract Eva software package, the crystallinity of the films was calculated. The crystallinity of the blank sample (CO‐1) was 23.9%, while the BSOR‐added samples (CO‐2 and CO‐3) exhibited 16.4% and 14.2% crystallinities, respectively. These results indicate that the addition of BSOR led to an increase in the amorphous nature of the film matrix and a decrease in crystallinity. Furthermore, a characteristic peak was consistently observed at a theta position of 20 degrees in all three samples. The slight variation in peak position and intensity can be attributed to the presence of BSOR. Previous studies conducted by other researchers also reported similar XRD patterns for these polymers (Dai et al., 2018; Dong et al., 2006; Thu & Ng, 2013).

## 4 CONCLUSION

In conclusion, this study explored the incorporation of BSOR into cellulose, sodium alginate, and gelatin composite edible films to evaluate its potential applications in food packaging. The results revealed that BSOR addition increased the film thickness, WVP, and yellow color intensity while reducing TS and EAB. However, the most significant finding was the enhancement of the films’ antioxidant properties with the inclusion of BSOR. These results underscore the potential of BSOR as a natural and effective antioxidant agent in edible film development, opening promising opportunities for its application in food packaging to extend shelf life and enhance food quality. Further research could explore optimizing BSOR concentrations and film formulations tailored to specific food packaging requirements.

## AUTHOR CONTRIBUTIONS

**Saurabh Bhatia:** Conceptualization (equal); supervision (equal); writing – original draft (equal). **Yasir Abbas Shah:** Methodology (equal); software (equal); writing – original draft (equal); writing – review and editing (equal). **Ahmed Al‐Harrasi:** Supervision (equal). **Sana Ullah:** Software (equal); writing – review and editing (equal). **Md Khalid Anwer:** Formal analysis (equal); validation (equal). **Esra Koca:** Formal analysis (equal); methodology (equal); software (equal). **Levent Yurdaer Aydemir:** Formal analysis (equal); methodology (equal); writing – review and editing (equal). **Mahbubar Rahman Khan:** Writing – review and editing (equal).

## FUNDING INFORMATION

The study was supported by TRC grant numbers BFP/GRG/HSS/22/106,l BFP/RGP/HSS/22/007 and MoHERI/SRPP/MoAFWR/1/2022/RI/01.

## CONFLICT OF INTEREST STATEMENT

The authors declare no conflicts of interest.

## INFORMED CONSENT STATEMENT

Not applicable.

## Data Availability

Even though adequate data has been given in the form of tables and figures, all authors declare that if more data is required, the data will be provided on a request basis.
